# Notes and News

**Published:** 1897-09-25

**Authors:** 


### NOTES AND NEWS.

(Collected and Communicated.)

On Saturday last an international conference of the Royal Red Cross Society was opened at Vienna.

The Middlesex Hospital has received a further sum of £2,000 from the estate of the late Mr. David Brandon.

"Lloyd's Newspaper" has taken up the cause of the Queen's Jubilee Hospital; and, as *Truth* points out, has thereby incurred a grave responsibility. Working men are likely to accept, without further question, the verdict of a paper which they so largely patronise.

A form of fever as yet unspecified has attacked the village of Stoke St. Michael, in the Radstock mining district, with fatal effect; the schoolmaster's family has been especially attacked, and the schools have been closed.

One of the disadvantages of the small number of entries for Army Medical Service, and the consequent inadequacy of the staff, which those who do enter will have to face in the future, will be the uncertainty of leave which must accrue when any call is made upon the resources of the department. Already the Indian section is feeling the pressure, and complaints are rife accordingly.

The New General Hospital at Birmingham has just been thrown open for public inspection. Over 6,000 admission tickets were issued, and 3,500 visited it the first day. There is still much to be done before the institution is in full working order. For instance, the electric installation is not yet completed. Some of the furniture from the old general hospital will be utilised. Already 120 new patients are waiting admission, the numbers at the old hospital having been reduced to a minimum. The public are so clamorous to see the magnificent new buildings that the management may find themselves obliged to extend the time they had fixed for their admission.

Through the will of the late Lady Jane Dundas the Royal Infirmary, Edinburgh, is to receive £5,000.

It is reported that opium eating has become a habit amongst the Kaffirs. The Chinese are said to be the chief purveyors, though the trade is by no means restricted to them.

One of the features of the proceedings at the Congress of the Sanitary Institute, held at Leeds last week, was a women's conference on the health work of women. Mrs. Fawkes, of Farnley Hall, presided.

Mr. Victor Horsley has consented to be nominated for the vacant seat as a direct representative on the General Medical Council, vacant through Dr. Rentoul's retirement. The poll will be declared on October 15th or 16th. The final day for receiving nomination papers is September 27th, and voting commences on October 2nd.

The Belfast Corporation have recommended the free gift of a site of six acres in the Asylum grounds for the erection of the new Royal Victoria Hospital. The site fulfils the conditions considered necessary by the Hospital Scheme Committee—it is salubrious, spacious, and near the populated quarter whence patients will be drawn. Moreover, the suggestion appears to meet with general approval.

We learn that the Queen has decided that the gifts and addresses presented to her Majesty in commemoration of the sixtieth anniversary of her reign shall be publicly exhibited at the Imperial Institute, and that one-half of the proceeds of their exhibition shall be presented to the Prince of Wales's Hospital Fund. The exhibition will probably commence about the middle of October next.

A case has recently come before the Courts which reveals the difficult position in which a poor law medical officer may be placed when reporting to his Board of Guardians. The case we allude to was that of the medical officer at Chiswick, who made a report in defence of his action in respect to some poor patients, concerning which the Board questioned him. The report contained some strong statements regarding the parents of the patients, and as it was read publicly before the Board, with reporters present, he was sued for libel by them. The defence that the doctor believed the statements to be true, and that he made them without malicious intention, did not avail with the jury before whom the case was tried, and £200 damages was awarded. The Judge, stating his opinion that the award was excessive, gave permission for the case to be retried, and advised the parents to accept a less sum. The case as it stands seems a hard one; but, putting on one side altogether the particular case in question, a decision of this sort seems likely to add greatly to the difficulties of poor law guardians in the administration of poor relief. If communications between them and their paid officers concerning their work are not to be considered privileged; if, for example, the reports of relieving officers as to the character of the applicants are to be taken as giving ground for actions for libel, we fail to see how guardians are to perform their duties.

